# Unexpected complication following total thyroidectomy: A case report and brief review of the literature

**DOI:** 10.3892/mi.2026.302

**Published:** 2026-02-12

**Authors:** Aso N. Qadir, Shaho F. Ahmed, Farman J. Ahmed, Bootan Jasim Raheem, Twana Omer Saeed, Ashna Othman Hussein, Zana Hama Amin, Diya Tahir Qadir, Berun A. Abdalla, Fahmi H. Kakamad

**Affiliations:** 1 Department of Scientific Affairs, Smart Health Tower, Sulaymaniyah 46001, Iraq; 2 Department of Endocrinology, Shar Hospital, Sulaymaniyah 46001, Iraq; 3 Kscien Organization for Scientific Research (Middle East Office), Sulaymaniyah 46001, Iraq; 4 College of Medicine, University of Sulaimani, Sulaymaniyah 46001, Iraq

**Keywords:** supraventricular tachycardia, hypothyroidism, post-thyroidectomy, atrioventricular nodal reentrant tachycardia, palpitations

## Abstract

Supraventricular tachycardia (SVT) involves a group of supraventricular tachyarrhythmiasfast heart rhythms originating above the ventricles. While SVT is commonly associated with hyperthyroidism, its occurrence in hypothyroidism is rare. The present case report describes a case of SVT occurring in the setting of post-thyroidectomy hypothyroidism. A 50-year-old female patient with a history of total thyroidectomy presented with palpitations and chest tightness. A 12-lead electrocardiogram (ECG) demonstrated atrioventricular nodal reentrant tachycardia (AVNRT) with a heart rate of 200 bpm. A laboratory evaluation revealed elevated thyroid-stimulating hormone levels, indicating biochemical hypothyroidism. Adenosine was administered, successfully restoring sinus rhythm. She was discharged with a revised levothyroxine regimen. At the 2-month follow-up, she remained asymptomatic, hemodynamically stable and free of recurrent arrhythmia. Ventricular and supraventricular arrhythmias, including SVT and Torsades de Pointes, are uncommon in hypothyroidism. The most common underlying mechanism is re-entry, with AVNRT being most common. In addition, the present study reviewed 4 cases of SVT following thyroidectomy reported in the literature. Palpitations were universal, and dyspnea occurred in 3 cases. In total, 2 patients were on hypothyroid treatment at the time, and similarly, 2 patients received adenosine intravenously. Myocardial structural changes, and slow cardiac repolarization lead to early after-depolarizations. A review of case reports revealed SVT occurring during hypothyroid states, particularly in women, often presenting with palpitations and diagnosed via ECG. Management relies on ECG-guided diagnosis and acute treatment with vagal maneuvers or adenosine. The identification of SVT in hypothyroidism is rare, but should be considered. ECG is key for diagnosis and management.

## Introduction

Supraventricular tachycardia (SVT) refers to a diverse group of cardiac arrhythmias, characterized by abnormally rapid supraventricular rhythms originating at or above the level of the bundle of His (1). These rhythms are defined by heart rates >100 bpm and involve mechanisms arising from atrial tissue and the atrioventricular junction. This group encompasses atrioventricular nodal reentrant tachycardia (AVNRT), atrial tachycardia (AT) (both focal and multifocal forms), several types of accessory pathway-related atrioventricular reentrant tachycardias (AVRT), atrial flutter, atrial fibrillation and sinus tachycardia (2). The incidence of SVT is ~0.225 per 100 individuals, with a female predominance, occurring almost twice as often in women across all age groups (1). Thyroidal hormones are essential for maintaining normal cardiac and vascular function (3). Thyroid irregularities affect ~10 to 15% of adult women and a smaller proportion of adult men (4). Thyroid disorders, including both hypothyroidism and hyperthyroidism, are linked to a higher risk of developing cardiovascular conditions.

Hyperthyroidism can cause both cardiac and hemodynamic symptoms, including rapid heartbeat, palpitations, atrial fibrillation, shortness of breath during physical activity, and reduced exercise capacity. The symptoms of hypothyroidism on the cardiovascular system are often more subtle and may include bradycardia, reduced pulse pressure and diastolic hypertension (5). SVT is commonly linked to hyperthyroidism, while its occurrence due to hypothyroidism is unusual (6). The present case report presents a rare case of SVT following thyroidectomy in the setting of biochemical hypothyroidism, as well as a brief literature review.

## Case report

### Patient information

On July 1, 2025, a 50-year-old female patient presented to Smart Health Tower (Sulaymaniyah, Iraq) with palpitations and a sensation of chest tightness that began ~1 h prior to presentation. She denied having chest pain, shortness of breath, orthopnea or other associated symptoms. Her medical history included a total thyroidectomy performed 3 years prior, and was receiving levothyroxine 100 mcg daily. She had also undergone axillary lymph node dissection and chemotherapy 12 years earlier; however, the specific treatment details were unavailable. She had previously been prescribed metoprolol at 50 mg empirically for intermittent palpitations, without a prior confirmed diagnosis of arrhythmia or structural heart disease. She had no history of hypertension, diabetes mellitus, asthma, or known cardiac disease. No known drug allergies were reported.

### Clinical findings

The patient appeared anxious but alert, with no signs of respiratory distress. Vital signs revealed a rapid, regular heart rate of ~200 bpm. Cardiac auscultation demonstrated normal first and second heart sounds (S1 and S2), with no murmurs, gallops, or additional heart sounds. Peripheral pulses were weak in volume.

The assessment for heart failure revealed mildly elevated jugular venous pressure, clear lung fields bilaterally on auscultation and no hepatomegaly. A lower extremity examination revealed mild peripheral edema that was subtle and not prominent, with no signs of significant fluid overload.

As regards hypothyroid-related features, the patient reported mild weight gain and cold intolerance. A physical examination revealed dry skin, with no facial puffiness. Deep tendon reflexes were normal, without delayed relaxation.

Overall, the findings of the physical examination were consistent with SVT in the absence of overt heart failure, accompanied by mild clinical features suggestive of hypothyroidism.

### Diagnostic approach

**Figure 1:**
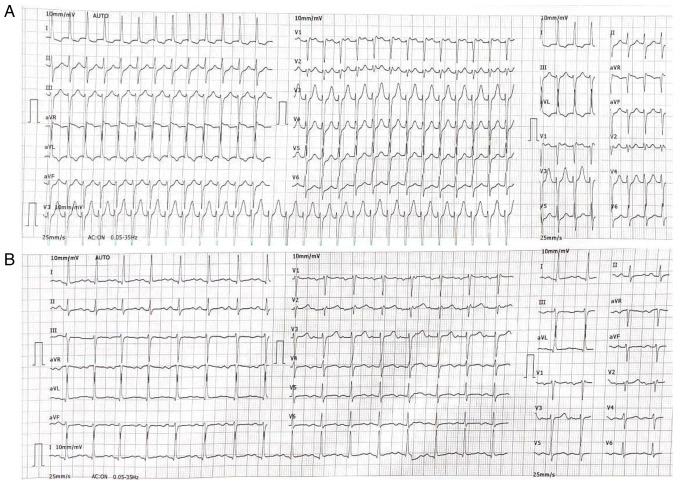
ECG results. (A) Upon administration, the ECG revealed a regular, narrow-complex tachycardia with a heart rate of ~160-180 bpm. P-waves are not clearly visible, likely hidden within or just after the QRS complexes. The QRS duration is narrow (<120 msec). These findings are consistent with paroxysmal supraventricular tachycardia, most likely atrioventricular nodal reentrant tachycardia, given the regular rhythm, narrow QRS complexes, and absent or retrograde P-waves. (B) Following the administration of 6 mg adenosine, the ECG revealed a normal sinus rhythm with a heart rate of ~75-85 bpm. P-waves are clearly visible and upright in lead II, consistent with sinus rhythm, and the QRS duration remains normal. ECG, electrocardiogram.

A 12-lead electrocardiogram (ECG) demonstrated a regular, narrow QRS complex tachycardia at approximately 200 bpm, with absent visible P-waves and a short RP interval, findings consistent with typical AVNRT (Fig. 1). Laboratory investigations revealed a complete blood count within normal limits, with a white blood cell count of 9.6x10^9^/l, hemoglobin level of 14.9 g/dl, hematocrit of 44.0%, red blood cell count of 5.04x10^12^/l, a mean corpuscular volume of 87.3 fl, mean corpuscular hemoglobin of 29.7 pg, mean corpuscular hemoglobin concentration of 34.0 g/dl, and a platelet count of 230x10^9^/l. The differential count revealed lymphocytes at 37.1%, granulocytes at 58.0%, and mid-sized cells at 4.9%, all within normal reference ranges. There were no notable findings in the levels of inflammatory markers, with a C-reactive protein level of 2.66 mg/l (reference range, <5.0 mg/l). Serum electrolytes were within normal limits, including sodium 143.1 mmol/l (reference range, 135-145 mmol/l), potassium 3.87 mmol/l (reference range, 3.5-5.1 mmol/l) and chloride 103.4 mmol/l (96-106 mmol/l). Thyroid function testing revealed an elevated thyroid-stimulating hormone (TSH) level of 17.7 µIU/ml, while free thyroxine (T4) remained within the normal range at 14.5 pmol/l. Transthoracic echocardiography demonstrated (images are not available; the institutional system is not directly integrated with the Picture Archiving and Communication System; therefore, the echocardiography images for this patient were not archived and could not be retrieved) normal cardiac chamber dimensions, preserved left ventricular systolic function and no structural or valvular abnormalities.

### Therapeutic intervention

Given her history of neck surgery and concerns about the effectiveness of vagal maneuvers, 6 mg of adenosine was administered as a rapid intravenous bolus. Successful rhythm conversion was achieved, with the prompt resolution of symptoms. The patient was discharged with an adjusted levothyroxine regimen based on the elevated TSH level, aimed at achieving biochemical euthyroidism, consisting of 100 mcg daily with an additional 50 mcg twice weekly.

### Follow-up and outcome

At the 2-month follow-up, the patient remained asymptomatic with no recurrence of symptoms. She was hemodynamically stable throughout follow-up period, and no episodes of arrhythmia were reported.

## Discussion

Ventricular and supraventricular arrhythmias are rarely associated with hypothyroidism, such as SVT and Torsades de Pointes. Hypothyroidism presents with a range of cardiovascular effects, which vary according to the severity and duration of the condition. These effects can range from mild changes to clearly noticeable symptoms. Common symptoms of SVT include palpitations, dizziness, chest discomfort, a pounding sensation in the neck or chest, and dyspnea. The primary electrophysiological mechanism underlying SVT is re-entry, whereas less frequent mechanisms include abnormal automaticity and triggered activity (4). Paroxysmal SVT, which encompasses arrhythmias such as AVRT, AVNRT and AT refers to a specific group of SVTs characterized by sudden initiation and termination (2). SVT may contribute to increased morbidity, particularly with persistent or frequent symptoms. In rare cases involving concomitant atrial fibrillation and ventricular pre-excitation, the condition may be potentially life-threatening (1).

Several similar cases of SVT occurring in the setting of hypothyroidism were reviewed (Table I) (4,6-8). The review included 4 cases, all of whom were female, with only 1 patient being >40 years of age. Palpitations were reported in all cases, while dyspnea was observed in 3 cases. An ECG was used in all cases to establish the diagnosis. Of note, 2 patients were receiving treatment for hypothyroidism at the time of SVT diagnosis, and 2 patients were treated with intravenous adenosine, similar to the present case. Notably, predisposing cardiac history was not consistently reported in the reviewed cases, which limiting the assessment of underlying cardiac susceptibility to SVT. The occurrence of tachyarrhythmias in hypothyroid states has been attributed to several proposed mechanisms, such as changes in genes specific to cardiac muscle cells, interstitial fluid accumulation, swelling of myofibrils with loss of normal striations, increased arterial rigidity, dysfunction of the endothelium, early onset of atherosclerosis, imbalances in autonomic regulation, with a shift toward heightened sympathetic activity, and autoimmunity. In general, hypothyroidism is deemed to cause an imbalance in autonomic nervous system control, marked by reduced modulation from both sympathetic and parasympathetic pathways, with a relative predominance of sympathetic activity (4).

Electrocardiographic abnormalities associated with hypothyroidism commonly include sinus bradycardia, low voltage QRS complexes, delayed electrical conduction, atrioventricular or bundle branch blocks, and, less commonly, a prolonged QT interval and Torsades de Pointes (7,9). The mechanisms underlying QT prolongation and Torsades de Pointes in hypothyroid patients may also provide insight into the development of paroxysmal SVT in this condition. During phase one of the cardiac action potential, depolarization occurs as a result of rapid sodium and calcium influx. In hypothyroidism, the slow inward current is reduced, particularly during phases two and three, predisposing to single or repetitive depolarizations known as early afterdepolarizations (10). These may manifest as abnormal U-waves on the ECG, and if they reach a certain threshold, they can trigger various forms of tachyarrhythmia. Such phenomena are more prevalent in the deep endocardium and mid-myocardial M-cell layers due to reduced delayed rectifier potassium currents (9). A previous study evaluating the effect of thyroid hormone replacement on benign atrial and ventricular arrhythmias found that thyroxine therapy was associated with a higher occurrence of atrial premature complexes in patients who already had existing arrhythmia. However, it did not lead to the development of new supraventricular or ventricular tachyarrhythmias. Nevertheless, the potential contribution of thyroxine therapy to tachyarrhythmogenesis cannot be entirely excluded (11).

Palpitations are commonly experienced by individuals at some point; however, not all palpitations are caused by arrhythmias, and not all arrhythmias result in palpitations. In some cases, arrhythmias can be diagnosed using ECG, whereas in others, diagnosis is based primarily on reported symptoms. In such situations, electrophysiological studies may help confirm the presence and elucidate the underlying mechanism of the abnormal cardiac rhythm (12). Thyroid dysfunction, particularly hyperthyroidism, is a recognized cause of palpitations. The association between atrial fibrillation and thyroid abnormalities is well established. By contrast, the association between thyroid dysfunction and other supraventricular tachyarrhythmias (excluding atrial fibrillation) is less clearly defined. Current guidelines recommend evaluation for hyperthyroidism in cases of inappropriate sinus tachycardia or frequent premature beats (13).

The clinical significance of SVT is not limited to adults, as it can also occur in the fetal and pediatric populations, where it may lead to serious complications such as fetal heart failure and hydrops fetalis, a potentially life-threatening condition. In children, SVT is primarily caused by three electrophysiological mechanisms, with re-entry circuits being the most common, followed by triggered activity and abnormal automaticity, all of these mechanisms may produce similar ECG patterns. In addition, SVT may be associated with structural heart defects, electrolyte disturbances and genetic factors (14).

Careful analysis of the surface ECG can identify features suggestive of the underlying mechanism in ~80% of cases of AVRT and AVNRT, although comparison with a sinus rhythm ECG is often helpful. AVNRT is the most common form of SVT in the general population, representing >60% of cases evaluated by invasive cardiac electrophysiological studies. This arrhythmia is caused by the presence of two functionally distinct conduction pathways within the atrioventricular node, one fast and one slow, that differ in conduction velocity and refractory period. AVNRT is typically initiated by an atrial premature beat, and less commonly by a ventricular premature beat, which establishes a sustained re-entrant circuit between these pathways. On a surface ECG, this manifests as a narrow complex tachycardia without signs of bundle branch block (1).

When the initiating premature impulse conducts antegradely through the slow pathway, a prolonged PR interval is observed the first beat of the tachycardia. During the re-entrant circuit, simultaneous activation of the atria and ventricles occurs, causing the P-wave and QRS complex to appear almost simultaneously. This leads to the absence of a visible RP interval or the presence of a very short RP interval, characteristic of typical AVNRT. The P-wave typically appears inverted (negative) in leads II, III, and augmented vector foot (aVF) due to retrograde atrial activation from the lower to the upper atrium. In addition, the P-waves are narrow, reflecting septal atrial activation (2).

The acute management of regular narrow complex tachycardia focuses on terminating the arrhythmia and identifying the underlying mechanism. A 12-lead ECG is essential for diagnosis and for guiding treatment decisions. In hemodynamically stable patients, vagal maneuvers may be attempted as an initial management strategy, with reported success rates of 19-54% when performed using a modified Valsalva technique (1,15), with improved results in a semi-recumbent position as per the REVERT trial (15). In the event that vagal maneuvers are unsuccessful or cannot be performed, adenosine may be administered as a rapid intravenous bolus through a large vein, followed immediately by a 10-ml saline flush. Due to its short half-life (<10 sec), rapid delivery is crucial. Adenosine functions by transiently slowing or blocking AV nodal conduction through activation of A1 receptors. Continuous ECG monitoring is essential during administration. If there is no response, the adenosine dose may be insufficient, or the rhythm may be ventricular tachycardia, which doesn't involve the AV node. The abrupt termination of the tachycardia following adenosine administration suggests AVNRT or AVRT, typically requiring ~6 mg. The presence of visible P-waves with AV dissociation is more consistent with AT. Adenosine rarely terminates AT; however, if it does, a focal origin is more likely than a re-entrant one. In patients who are hemodynamically unstable, immediate direct-current cardioversion is indicated, immediate direct current cardioversion is indicated, though adenosine may be attempted during preparation (1).

The present case report has several key considerations. First, collecting data from multiple patients with post-thyroidectomy biochemical hypothyroidism and AVNRT is challenging, which limits the feasibility of larger studies. Second, although the patient had been receiving metoprolol for several years, her arrhythmic signs and symptoms developed only recently, suggesting the presence of additional contributing factors, such as hormonal fluctuations. Third, the prior years of hypothyroidism treatment of the patient were reviewed as part of the data collection in order to better understand long-term thyroid management. Finally, the primary focus of the present report was the association between levothyroxine dose adjustment and arrhythmia, as the condition of the patient improved the 6 six months following the modification of thyroid hormone therapy, highlighting the potential impact of optimized dosing on arrhythmia control.

In conclusion, the occurrence of SVT in patients with hypothyroidism is rare, but should still be considered by clinicians. ECG plays a crucial role in both the diagnosis and management of this condition.

## Figures and Tables

**Table I:** Review of some cases of supraventricular tachycardia with hypothyroidism identified in the literature.

Authors, year of publication	Age	Sex	Clinical findings	On medication	Thyroid function test	Electrocardiogram findings	Treatment	Outcome	(Refs.)
Maznun *et al*, 2024	28	F	Palpitations, dyspnea and underlying hypothyroidism.	Levothyroxine.	Revealed hypothyroidism.	Revealed SVT.	Carotid massage and levothyroxine.	In a stable condition.	(6)
Mak *et al*, 2018	26	F	Palpitations, profuse sweating and presyncopal attack.	None.	Severe hypothyroidism, free T4: 0.3 pmol/l, TSH: 100 mIU/ml TPO IgG: 101 IU/ml.	Revealed SVT.	IV adenosine and thyroxine replacement therapy.	In a stable condition.	(4)
Tomar *et al*, 2015	32	F	Palpitations and dyspnea associated with diaphoresis.	Oral levothyroxine.	TSH: 44.3 µU/ml, free T3: 2.3 pg/ml, free T4: 0.3 ng/ml, TPO IgG: +ve.	Revealed paroxysmal SVT.	Carotid massage initially, IV adenosine 12 mg due to persistent tachyarrhythmia, thyroxine and diltiazem.	In a stable condition.	(7)
Olarescu *et a*l, 2009	47	F	Palpitation, dyspnea, dizziness and anxiety.	Not mentioned.	Free T4: 11.25 pmol/l, TSH=26.37 µUI/ml, TPO IgG: 1/640.	Revealed paroxysmal SVT.	Thyroxin replacement therapy.	In a stable condition.	(8)

M, male; F, female; SVT, supraventricular tachycardia; TSH, thyroid-stimulating hormone; TPO IgG, thyroid peroxidase IgG antibody, +ve, positive; IV, intravenous.

## Data Availability

The data generated in the present study may be requested from the corresponding author.
